# Crystal structure of [4-(2-meth­oxy­phen­yl)-3-methyl-1-phenyl-6-tri­fluoro­methyl-1*H*-pyrazolo­[3,4-*b*]pyridin-5-yl](thio­phen-2-yl)methanone

**DOI:** 10.1107/S1600536814017437

**Published:** 2014-08-06

**Authors:** V. Rajni Swamy, P. Gunasekaran, R. V. Krishnakumar, N. Srinivasan, P. Müller

**Affiliations:** a Department of Physics, Thiagarajar College, Madurai 625 009, India; b School of Chemistry, Madurai Kamaraj University, Madurai 625 021, India; c X-Ray Diffraction Facility, MIT Department of Chemistry, 77 Massachusetts Avenue, Building 2, Room 325, Cambridge, MA, 02139-4307, USA

**Keywords:** crystal structure, 1*H*-pyrazolo­[3,4-*b*]pyridine, hydrogen bonding graph-set analysis

## Abstract

The title compound, C_26_H_18_F_3_N_3_O_2_S, a 2-meth­oxy-substituted derivative, is closely related to its 4-methyl- and 4-chloro-substituted analogues and yet displays no structural relationships with them. The thio­phene ring is disorder free and the –CF_3_ group exhibits disorder, respectively, in contrast and similar to that observed in the 4-methyl- and 4-chloro-substituted derivatives. The torsion angle which defines the twist of the thio­phene ring is −69.6 (2)° (*gauche*) in the title compound, whereas it is anti­clinal in the 4-methyl- and 4-chloro-substituted derivatives, with respective values of 99.9 (2) and 99.3 (2)°. The absence of disorder in the thio­phene ring facilitates one of its ring C atoms to participate in the lone inter­molecular C—H⋯O hydrogen bond present in the crystal, leading to a characteristic *C*(5) chain graph-set motif linking mol­ecules related through glides along [010]. An intra­moleculr C—H⋯N hydrogen bond also occurs.

## Related literature   

For the biological activity of 1*H*-pyrazolo­[3,4-*b*]pyridines, see: Hardy (1984 ▶); Chu & Lynch (1975 ▶); Ali (2009 ▶); Wilson *et al.* (2013 ▶); Souza *et al.* (2012 ▶). For applications of thio­phene ring systems in solar cells, see: Hara *et al.* (2003 ▶). For related structures, see: Rajni Swamy *et al.* (2013 ▶). For the treatment of disorders in crystal structures, see: Müller (2009 ▶). For hydrogen-bond graph-set motifs, see: Bernstein *et al.* (1995 ▶).
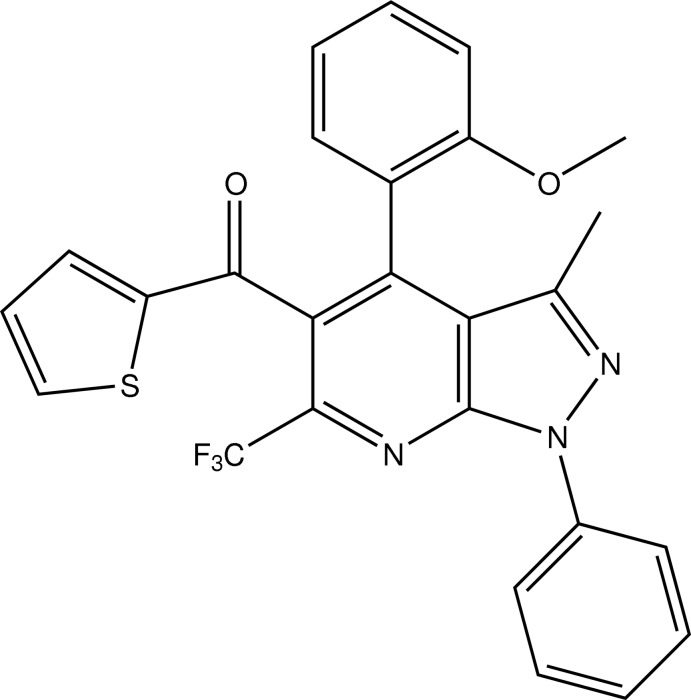


## Experimental   

### Crystal data  

C_26_H_18_F_3_N_3_O_2_S
*M* *_r_* = 493.49
Monoclinic, 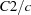

*a* = 18.9343 (11) Å
*b* = 11.6347 (6) Å
*c* = 20.9800 (12) Å
β = 92.511 (2)°
*V* = 4617.3 (4) Å^3^
*Z* = 8
Mo *K*α radiation
μ = 0.19 mm^−1^
*T* = 301 K
0.25 × 0.16 × 0.12 mm

### Data collection  

Bruker SMART APEXII CCD diffractometer
Absorption correction: multi-scan (*SADABS*; Bruker, 2009 ▶) *T*_min_ = 0.949, *T*_max_ = 0.979
23854 measured reflections
5492 independent reflections
3784 reflections with *I* > 2σ(*I*)
*R*_int_ = 0.039

### Refinement  

*R*[*F* ^2^ > 2σ(*F* ^2^)] = 0.048
*wR*(*F* ^2^) = 0.138
*S* = 1.02
5492 reflections
346 parameters
142 restraints
H-atom parameters constrained
Δρ_max_ = 0.33 e Å^−3^
Δρ_min_ = −0.47 e Å^−3^

### 

Data collection: *APEX2* (Bruker, 2009 ▶); cell refinement: *SAINT* (Bruker, 2009 ▶); data reduction: *SAINT*; program(s) used to solve structure: *SHELXS2013* (Sheldrick, 2008 ▶); program(s) used to refine structure: *SHELXL2013* (Sheldrick, 2008 ▶); molecular graphics: *PLATON* (Spek, 2009 ▶); software used to prepare material for publication: *SHELXL2013*.

## Supplementary Material

Crystal structure: contains datablock(s) I. DOI: 10.1107/S1600536814017437/hg5397sup1.cif

Structure factors: contains datablock(s) I. DOI: 10.1107/S1600536814017437/hg5397Isup2.hkl

Supporting information file. DOI: 10.1107/S1600536814017437/hg5397Isup3.cml

**Fig. 1.**
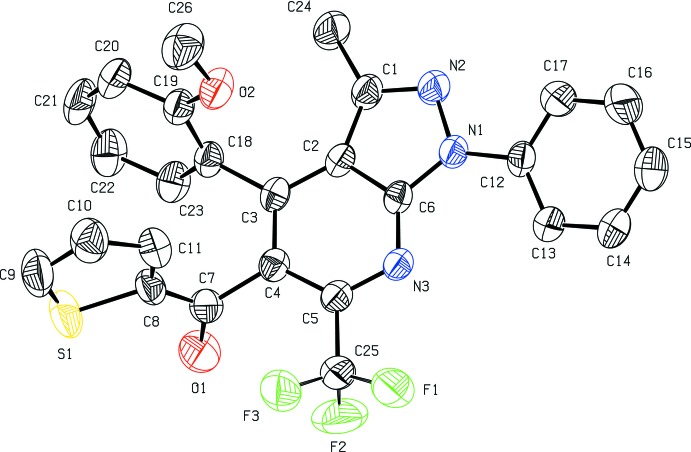
Molecular structure of (I) showing the atom numbering scheme and displacement ellipsoids drawn at the 50% probability level. H atoms and atoms of minor disorder components have been omitted for clarity.

. DOI: 10.1107/S1600536814017437/hg5397fig1.tif
Mol­ecular structure of (I) showing the atom numbering scheme and displacement ellipsoids drawn at the 50% probability level. H atoms and atoms of minor disorder components have been omitted for clarity.

**Fig. 2.**
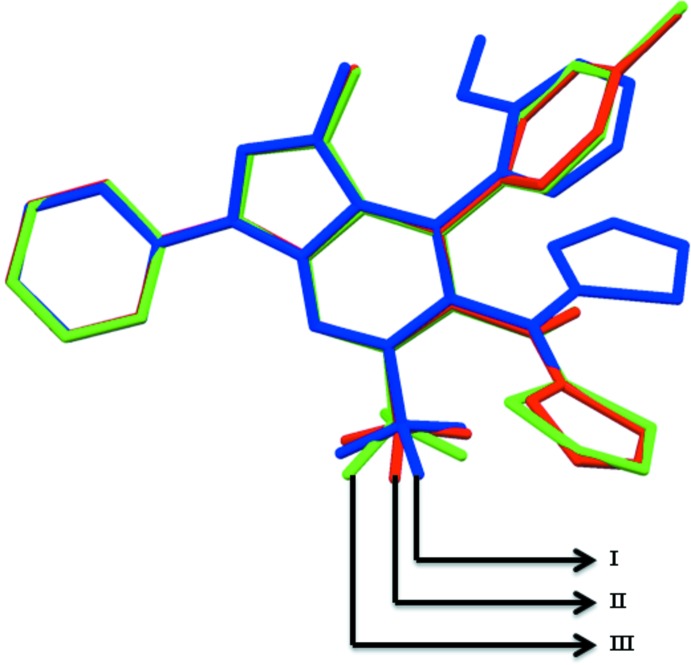
Overlay of the molecular structures of (I) (blue), 4- methyl (red) and 4-chloro (green) analogues. H atoms and atoms of minor disordered components were not included in the least squares fit of the atomic positions.

. DOI: 10.1107/S1600536814017437/hg5397fig2.tif
Overlay of the mol­ecular structures of (I) (blue), 4- methyl (red) and 4-chloro (green) analogues. H atoms and atoms of minor disordered components were not included in the least squares fit of the atomic positions.

**Fig. 3.**
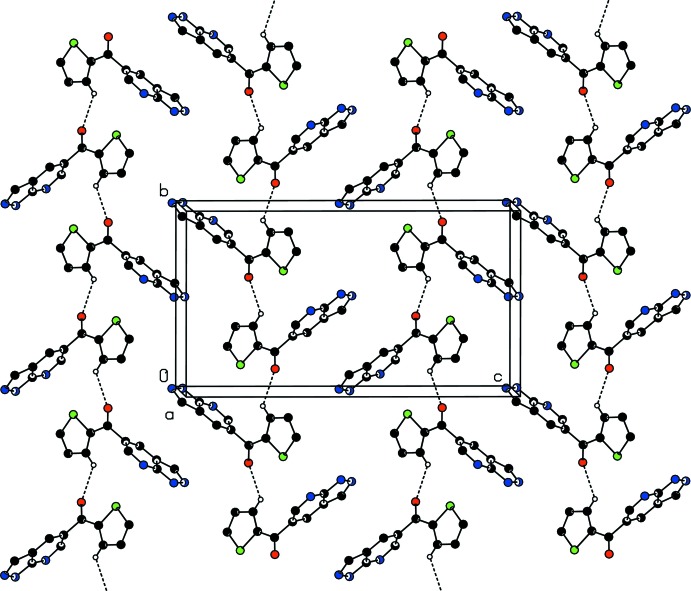
C—H···O hydrogen bond linking molecules through chains extending infinitely along [010]. Non-participating ring atoms and groups have been omitted for clarity.

. DOI: 10.1107/S1600536814017437/hg5397fig3.tif
C—H⋯O hydrogen bond linking mol­ecules through chains extending infinitely along [010]. Non-participating ring atoms and groups have been omitted for clarity.

CCDC reference: 1016829

Additional supporting information:  crystallographic information; 3D view; checkCIF report

## Figures and Tables

**Table 1:** Hydrogen-bond geometry (Å, °)

*D*—H⋯*A*	*D*—H	H⋯*A*	*D*⋯*A*	*D*—H⋯*A*
C13—H13⋯N3	0.93	2.42	3.012 (2)	122
C11—H11⋯O1^i^	0.93	2.51	3.114 (3)	122

Symmetry code: (i) 
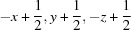
.

## supplementary crystallographic information

### S1. Comment

Many polysubstituted derivatives of 1*H*-pyrazolo­[3,4-*b*]pyridine have been synthesized as potentially biologically active materials (Hardy, 1984; Chu & Lynch, 1975) and established as anti­microbial agents against bacterial and fungal strain (Ali, 2009). Thio­phenes are regarded as important building units for a variety of drugs. Recently, a new class of thio­phene compounds that kill extensively drug resistant *Mycobacterium tuberculosis* have been reported (Wilson *et al.*, 2013). An evaluation of the cytotoxic activities of some thio­phene derivatives have indicated that they could be considered promising compounds for the discovery of new anti­tumor agents (Souza *et al.*, 2012). Also, in the background of coumarin dyes being successfully used as organic dye photo-sensitizers for Dye-Sensitized-Solar-Cells (DSSC), the design of new coumarin dyes through the introduction of thio­phene moieties have shown to remarkably improve the solar cell performance (Hara *et al.*, 2003).

The title compound, C_26_H_18_F_3_N_3_O_2_S, a 2-meth­oxy-substituted derivative, is closely related to its 4-methyl and 4-chloro analogues which have been earlier shown to obey the chloro-methyl exchange rule [Rajni Swamy *et al.*, 2013] and yet displays no structural relationships. The title compound may be regarded as an example of a case where change in the nature and position of the substitution may affect intra and inter molecular inter­actions which in turn might alter the molecular geometry quite noticeably. Fig.1 shows the molecular structure of (I) showing the atom numbering scheme and displacement ellipsoids drawn at the 50% probability level.

The bond lengths and angles are comparable in all the three structures with deviations evident in the torsion angles related to the thio­phene group which may be attributed to the absence of disorder in the ring, unlike in the methyl- and chloro- substituted derivatives. The torsion angle C3—C4—C7—C8 which defines the twist of the thio­phene ring is –69.6 (2)° [*gauche*] in the title compound whereas it is *anti­clinal* in 4-methyl and 4-chloro derivatives with respective values of 99.9 (2)° and 99.3 (2)° (Figure 2). Correspondingly, the orientation of the carbonyl O with respect to the carbon to which the CF_3_ group is attached, defined by the torsion angle C5—C4—C7—O1 is –70.8 (3)° in the present compound and 98.6 (2)° and 98.8 (2)°, respectively for 4-chloro and 4-methyl analogues.

The intra­molecular distances H13···N3 and H17···N2, on either sides of the phenyl ring, involving the N3 and N2 atoms of the pyrazolo-pyridine rings are 2.416 (1)Å and 2.436 (2) Å respectively. The increased values observed for these distances in 4-methyl and 4-chloro counterparts are 2.483 (1)Å and 2.513( 1)Å which is due to the participation of the phenyl ring (atom C15) in an inter­molecular C—-H···.O hydrogen bond. The shortening of these distances in the present case is correlated to the non-participation of the phenyl ring in the hydrogen bond, which in turn has caused the associated phenyl and the fused pyrazolo-pyridine ring systems tend towards coplanarity. Thus, a C13—H13···N3 intra­molecular hydrogen bond may well be regarded as present. The same argument is invalid for considering C17—H17···N2 as a hydrogen bond since its geometry is more of sterical consequence rather than a hydrogen bonded requirement.

The absence of disorder in the thio­phene ring facilitates one of its ring C11 atom to participate in the lone inter­molecular C—H···O hydrogen bond present in the crystal. As a consequene, the thio­phene ring deviates significantly from being perpendicular to the central fused pyrazolo-pyridine ring. The C11—H11···O1 inter­action gives rise to a characteristic C(5) chain graph-set motif [Bernstein *et al.*,1995] which links molecules related through glides extending along [010] (Figure 3). Thus, the scheme of non-covalent inter­actions is entirely different compared to the 4-methyl and 4-chloro analogues and the significant C—H···π inter­action observed in them is absent in the present case.

### S2. Refinement

The structure displays disorder of the CF_3_ group. The disorder was refined with the help of similarity restraints on 1-2 and 1-3 distances and displacement parameters as well as rigid bond restraints (*aka* Hirshfeld restraints) for anisotropic displacement parameters (Müller, 2009). The first approach to the CF_3_ disorder was to refine the CF_3_ group as freely rotating about the C5—C25 bond. The thermal ellipsoids of the six fluorine atoms form a circular toroid as expected for a pure rotation about the C—C bond, elongated in a direction approximately perpendicular to the aromatic ring plane to which the CF_3_ group binds. The occupancy ratio of the two components was refined freely and converged at 0.956 (3).

All hydrogen atoms except the nitro­gen bound hydrogens, were included into the model at geometrically calculated positions (C—H target distance 0.96Å for methyl hydrogen atoms, 0.93Å for all others) and refined using a riding model. The torsion angle of the methyl groups were allowed to refine. The *U_iso_* values of all hydrogen atoms were constrained to 1.2 times *U_eq_* (1.5 times for methyl H atoms) of the respective atom to which the hydrogen atom binds.

### S3. Synthesis and crystallization

A mixture of 4,4,4-tri­fluoro-1-(thio­phen-2-yl)butane-1,3-dione (0.222g, 1 mmol), 2-meth­oxy benzaldehyde (0.136g, 1 mmol), 3- methyl-1-phenyl-1H-pyrazol-5-amine (0.173g, 1 mmol) in the presence of L-proline (20 mol%) in ethanol (15 ml) was stirred at 60^o^C for 18 hrs. After completion of the reaction (TLC), the reaction mixture was extracted with ethyl acetate (2 x 40 ml) and the residue after removal of the solvent was chromatographed over silica gel (230–400 mesh) using petroleum ether-ethyl acetate mixture (4:1 v/v), which afforded pure product as a yellow solid; Yield 82%; mp 213 °C. Yellow coloured needles were obtained from a saturated solution of the solute in petroleum ether-ethyl acetate (4:1) mixture.

### Crystal data

**Table:**

C_26_H_18_F_3_N_3_O_2_S	*F*(000) = 2032
*M**_r_* = 493.49	*D*_x_ = 1.420 Mg m^−^^3^
Monoclinic, *C*2/*c*	Mo *K*α radiation, λ = 0.71073 Å
*a* = 18.9343 (11) Å	Cell parameters from 2784 reflections
*b* = 11.6347 (6) Å	θ = 2.1–28.0°
*c* = 20.9800 (12) Å	µ = 0.19 mm^−^^1^
β = 92.511 (2)°	*T* = 301 K
*V* = 4617.3 (4) Å^3^	Needle, yellow
*Z* = 8	0.25 × 0.16 × 0.12 mm

### Data collection

**Table:**

Bruker SMART APEXII CCD diffractometer	3784 reflections with *I* > 2σ(*I*)
Radiation source: fine-focus sealed tube	*R*_int_ = 0.039
ω and φ scans	θ_max_ = 28.0°, θ_min_ = 2.2°
Absorption correction: multi-scan (*SADABS*; Bruker, 2009)	*h* = −24→24
*T*_min_ = 0.949, *T*_max_ = 0.979	*k* = −13→15
23854 measured reflections	*l* = −27→27
5492 independent reflections	

### Refinement

**Table:**

Refinement on *F*^2^	142 restraints
Least-squares matrix: full	Hydrogen site location: inferred from neighbouring sites
*R*[*F*^2^ > 2σ(*F*^2^)] = 0.048	H-atom parameters constrained
*wR*(*F*^2^) = 0.138	*w* = 1/[σ^2^(*F*_o_^2^) + (0.0601*P*)^2^ + 3.2782*P*] where *P* = (*F*_o_^2^ + 2*F*_c_^2^)/3
*S* = 1.02	(Δ/σ)_max_ < 0.001
5492 reflections	Δρ_max_ = 0.33 e Å^−^^3^
346 parameters	Δρ_min_ = −0.47 e Å^−^^3^

### Special details

**Table:**

Geometry. All e.s.d.'s (except the e.s.d. in the dihedral angle between two l.s. planes) are estimated using the full covariance matrix. The cell e.s.d.'s are taken into account individually in the estimation of e.s.d.'s in distances, angles and torsion angles; correlations between e.s.d.'s in cell parameters are only used when they are defined by crystal symmetry. An approximate (isotropic) treatment of cell e.s.d.'s is used for estimating e.s.d.'s involving l.s. planes.

### Fractional atomic coordinates and isotropic or equivalent isotropic displacement parameters (Å^2^)

**Table:**

	*x*	*y*	*z*	*U*_iso_*/*U*_eq_	Occ. (<1)
O1	0.30751 (9)	0.60534 (13)	0.20858 (9)	0.0688 (5)	
O2	0.11454 (7)	0.93337 (12)	0.14944 (7)	0.0548 (4)	
N1	0.30027 (8)	1.00767 (14)	0.01120 (7)	0.0406 (4)	
N2	0.23413 (8)	1.00212 (16)	−0.01836 (7)	0.0499 (4)	
N3	0.36085 (7)	0.91418 (13)	0.10024 (7)	0.0389 (3)	
C1	0.19684 (10)	0.92676 (18)	0.01270 (9)	0.0455 (5)	
C2	0.23793 (9)	0.88016 (16)	0.06502 (8)	0.0373 (4)	
C3	0.22736 (9)	0.80476 (15)	0.11561 (8)	0.0370 (4)	
C4	0.28594 (9)	0.78276 (15)	0.15652 (8)	0.0375 (4)	
C5	0.35026 (9)	0.83882 (16)	0.14613 (9)	0.0392 (4)	
C25	0.41493 (11)	0.8173 (2)	0.18938 (11)	0.0554 (5)	
F1	0.46297 (8)	0.89725 (17)	0.18471 (9)	0.0956 (8)	0.958 (3)
F2	0.44581 (9)	0.71752 (16)	0.17541 (10)	0.0964 (7)	0.958 (3)
F3	0.39939 (8)	0.80892 (16)	0.25001 (7)	0.0786 (6)	0.958 (3)
F1A	0.4098 (16)	0.7207 (9)	0.2222 (10)	0.098 (8)	0.042 (3)
F2A	0.4215 (12)	0.9027 (11)	0.2288 (9)	0.089 (8)	0.042 (3)
F3A	0.4718 (10)	0.8067 (10)	0.1573 (10)	0.070 (7)	0.042 (3)
C6	0.30449 (9)	0.93290 (15)	0.06141 (8)	0.0359 (4)	
C7	0.27888 (10)	0.69865 (16)	0.21061 (10)	0.0449 (4)	
C8	0.23554 (11)	0.73371 (17)	0.26263 (9)	0.0455 (5)	
S1	0.20657 (4)	0.63331 (6)	0.31536 (3)	0.0750 (2)	
C9	0.16353 (14)	0.7348 (3)	0.35569 (11)	0.0755 (8)	
H9	0.1372	0.7189	0.3911	0.091*	
C10	0.17100 (13)	0.8413 (2)	0.33156 (10)	0.0654 (6)	
H10	0.1514	0.9071	0.3487	0.078*	
C11	0.21214 (11)	0.84097 (18)	0.27723 (9)	0.0510 (5)	
H11	0.2222	0.9065	0.2539	0.061*	
C12	0.34875 (9)	1.09282 (17)	−0.00852 (8)	0.0406 (4)	
C13	0.41925 (10)	1.08884 (18)	0.01057 (10)	0.0488 (5)	
H13	0.4367	1.0284	0.0357	0.059*	
C14	0.46389 (12)	1.1758 (2)	−0.00798 (11)	0.0596 (6)	
H14	0.5114	1.1741	0.0053	0.072*	
C15	0.43889 (13)	1.2640 (2)	−0.04558 (13)	0.0725 (7)	
H15	0.4694	1.3213	−0.0585	0.087*	
C16	0.36868 (14)	1.2678 (2)	−0.06417 (15)	0.0842 (9)	
H16	0.3515	1.3283	−0.0893	0.101*	
C17	0.32333 (12)	1.1824 (2)	−0.04591 (12)	0.0667 (7)	
H17	0.2757	1.1852	−0.0588	0.080*	
C18	0.15781 (10)	0.75027 (16)	0.12743 (8)	0.0400 (4)	
C19	0.10172 (10)	0.81792 (17)	0.14666 (9)	0.0440 (4)	
C20	0.03917 (11)	0.7667 (2)	0.16311 (11)	0.0573 (6)	
H20	0.0019	0.8116	0.1763	0.069*	
C21	0.03220 (12)	0.6485 (2)	0.15987 (12)	0.0641 (6)	
H21	−0.0099	0.6144	0.1710	0.077*	
C22	0.08657 (13)	0.5807 (2)	0.14044 (11)	0.0613 (6)	
H22	0.0812	0.5014	0.1380	0.074*	
C23	0.14961 (11)	0.63175 (17)	0.12456 (10)	0.0495 (5)	
H23	0.1868	0.5861	0.1119	0.059*	
C24	0.12313 (11)	0.8992 (2)	−0.01037 (11)	0.0665 (7)	
H24A	0.1138	0.9338	−0.0514	0.100*	
H24B	0.0904	0.9288	0.0193	0.100*	
H24C	0.1177	0.8174	−0.0138	0.100*	
C26	0.06586 (14)	1.0042 (2)	0.18061 (14)	0.0743 (7)	
H26A	0.0214	1.0044	0.1567	0.112*	
H26B	0.0840	1.0812	0.1835	0.112*	
H26C	0.0592	0.9751	0.2227	0.112*	

### Atomic displacement parameters (Å^2^)

**Table:**

	*U*^11^	*U*^22^	*U*^33^	*U*^12^	*U*^13^	*U*^23^
O1	0.0734 (11)	0.0386 (8)	0.0952 (12)	0.0074 (7)	0.0113 (9)	0.0132 (8)
O2	0.0483 (8)	0.0456 (8)	0.0721 (10)	−0.0016 (6)	0.0197 (7)	−0.0015 (7)
N1	0.0333 (8)	0.0520 (9)	0.0364 (8)	−0.0078 (7)	0.0016 (6)	0.0040 (7)
N2	0.0375 (9)	0.0686 (11)	0.0432 (9)	−0.0097 (8)	−0.0022 (7)	0.0082 (8)
N3	0.0315 (8)	0.0438 (9)	0.0415 (8)	−0.0028 (6)	0.0038 (6)	0.0008 (7)
C1	0.0367 (10)	0.0603 (12)	0.0394 (10)	−0.0101 (9)	0.0013 (8)	0.0003 (9)
C2	0.0309 (9)	0.0444 (10)	0.0371 (9)	−0.0054 (7)	0.0053 (7)	−0.0052 (7)
C3	0.0349 (9)	0.0358 (9)	0.0409 (9)	−0.0054 (7)	0.0076 (7)	−0.0058 (7)
C4	0.0372 (9)	0.0320 (9)	0.0437 (10)	−0.0018 (7)	0.0059 (7)	−0.0018 (7)
C5	0.0345 (9)	0.0388 (9)	0.0443 (10)	−0.0007 (7)	0.0032 (7)	−0.0006 (7)
C25	0.0414 (11)	0.0599 (13)	0.0644 (13)	−0.0066 (9)	−0.0044 (9)	0.0167 (10)
F1	0.0582 (10)	0.1105 (14)	0.1143 (15)	−0.0439 (10)	−0.0387 (9)	0.0573 (12)
F2	0.0649 (11)	0.0951 (13)	0.1280 (16)	0.0346 (9)	−0.0088 (10)	0.0119 (11)
F3	0.0646 (9)	0.1126 (15)	0.0571 (8)	−0.0194 (9)	−0.0157 (7)	0.0211 (8)
F1A	0.098 (17)	0.106 (11)	0.089 (18)	0.005 (15)	−0.005 (13)	0.060 (11)
F2A	0.052 (15)	0.118 (12)	0.093 (16)	0.008 (15)	−0.036 (11)	−0.029 (11)
F3A	0.025 (5)	0.099 (18)	0.084 (15)	−0.028 (11)	−0.011 (6)	−0.006 (11)
C6	0.0335 (9)	0.0407 (9)	0.0339 (8)	−0.0034 (7)	0.0068 (7)	−0.0035 (7)
C7	0.0422 (10)	0.0363 (10)	0.0560 (12)	−0.0066 (8)	−0.0014 (8)	0.0067 (8)
C8	0.0496 (11)	0.0415 (11)	0.0452 (10)	−0.0113 (8)	−0.0014 (8)	0.0121 (8)
S1	0.0858 (5)	0.0661 (4)	0.0737 (4)	−0.0167 (3)	0.0118 (3)	0.0319 (3)
C9	0.0747 (17)	0.104 (2)	0.0486 (13)	−0.0226 (15)	0.0098 (12)	0.0209 (13)
C10	0.0702 (16)	0.0812 (17)	0.0453 (12)	−0.0061 (13)	0.0086 (11)	−0.0028 (11)
C11	0.0610 (13)	0.0492 (12)	0.0433 (10)	−0.0064 (10)	0.0069 (9)	0.0045 (9)
C12	0.0378 (10)	0.0486 (11)	0.0359 (9)	−0.0071 (8)	0.0082 (7)	0.0004 (8)
C13	0.0396 (10)	0.0526 (12)	0.0546 (11)	−0.0029 (9)	0.0067 (9)	0.0088 (9)
C14	0.0400 (11)	0.0694 (15)	0.0698 (14)	−0.0124 (10)	0.0074 (10)	0.0104 (12)
C15	0.0577 (15)	0.0693 (16)	0.0910 (19)	−0.0201 (12)	0.0091 (13)	0.0257 (14)
C16	0.0652 (17)	0.0789 (18)	0.108 (2)	−0.0117 (14)	−0.0018 (15)	0.0485 (17)
C17	0.0458 (12)	0.0777 (16)	0.0758 (15)	−0.0090 (11)	−0.0058 (11)	0.0299 (13)
C18	0.0386 (10)	0.0442 (10)	0.0375 (9)	−0.0105 (8)	0.0044 (7)	−0.0020 (7)
C19	0.0399 (10)	0.0490 (11)	0.0433 (10)	−0.0076 (8)	0.0051 (8)	0.0008 (8)
C20	0.0391 (11)	0.0674 (15)	0.0664 (14)	−0.0090 (10)	0.0127 (10)	0.0022 (11)
C21	0.0473 (13)	0.0717 (16)	0.0743 (15)	−0.0273 (11)	0.0128 (11)	0.0011 (12)
C22	0.0650 (15)	0.0510 (13)	0.0682 (14)	−0.0282 (11)	0.0063 (11)	−0.0068 (11)
C23	0.0504 (12)	0.0463 (11)	0.0523 (11)	−0.0131 (9)	0.0070 (9)	−0.0079 (9)
C24	0.0428 (12)	0.0990 (19)	0.0567 (13)	−0.0215 (12)	−0.0099 (10)	0.0125 (12)
C26	0.0680 (16)	0.0596 (15)	0.0976 (19)	0.0103 (12)	0.0274 (14)	−0.0030 (13)

### Geometric parameters (Å, º)

**Table:**

O1—C7	1.215 (2)	C10—C11	1.408 (3)
O2—C19	1.366 (2)	C10—H10	0.9300
O2—C26	1.418 (3)	C11—H11	0.9300
N1—C6	1.366 (2)	C12—C13	1.378 (3)
N1—N2	1.374 (2)	C12—C17	1.378 (3)
N1—C12	1.424 (2)	C13—C14	1.385 (3)
N2—C1	1.316 (2)	C13—H13	0.9300
N3—C5	1.324 (2)	C14—C15	1.367 (3)
N3—C6	1.332 (2)	C14—H14	0.9300
C1—C2	1.425 (3)	C15—C16	1.370 (4)
C1—C24	1.492 (3)	C15—H15	0.9300
C2—C3	1.398 (2)	C16—C17	1.379 (3)
C2—C6	1.407 (2)	C16—H16	0.9300
C3—C4	1.396 (3)	C17—H17	0.9300
C3—C18	1.492 (2)	C18—C23	1.389 (3)
C4—C5	1.407 (2)	C18—C19	1.396 (3)
C4—C7	1.509 (3)	C19—C20	1.383 (3)
C5—C25	1.512 (3)	C20—C21	1.383 (3)
C25—F2A	1.295 (12)	C20—H20	0.9300
C25—F3A	1.301 (11)	C21—C22	1.373 (3)
C25—F1	1.308 (2)	C21—H21	0.9300
C25—F3	1.322 (3)	C22—C23	1.387 (3)
C25—F1A	1.324 (12)	C22—H22	0.9300
C25—F2	1.338 (3)	C23—H23	0.9300
C7—C8	1.452 (3)	C24—H24A	0.9600
C8—C11	1.364 (3)	C24—H24B	0.9600
C8—S1	1.7153 (18)	C24—H24C	0.9600
S1—C9	1.683 (3)	C26—H26A	0.9600
C9—C10	1.349 (4)	C26—H26B	0.9600
C9—H9	0.9300	C26—H26C	0.9600
			
C19—O2—C26	118.27 (16)	C8—C11—H11	123.8
C6—N1—N2	109.85 (14)	C10—C11—H11	123.8
C6—N1—C12	130.57 (15)	C13—C12—C17	120.05 (18)
N2—N1—C12	119.19 (15)	C13—C12—N1	121.39 (17)
C1—N2—N1	107.80 (15)	C17—C12—N1	118.53 (17)
C5—N3—C6	114.23 (15)	C12—C13—C14	119.3 (2)
N2—C1—C2	110.39 (16)	C12—C13—H13	120.4
N2—C1—C24	119.79 (18)	C14—C13—H13	120.4
C2—C1—C24	129.78 (18)	C15—C14—C13	120.7 (2)
C3—C2—C6	118.23 (16)	C15—C14—H14	119.6
C3—C2—C1	137.08 (16)	C13—C14—H14	119.6
C6—C2—C1	104.64 (15)	C14—C15—C16	119.7 (2)
C4—C3—C2	116.46 (15)	C14—C15—H15	120.2
C4—C3—C18	120.20 (16)	C16—C15—H15	120.2
C2—C3—C18	123.33 (16)	C15—C16—C17	120.4 (2)
C3—C4—C5	119.31 (16)	C15—C16—H16	119.8
C3—C4—C7	119.15 (15)	C17—C16—H16	119.8
C5—C4—C7	121.54 (16)	C12—C17—C16	119.8 (2)
N3—C5—C4	125.40 (17)	C12—C17—H17	120.1
N3—C5—C25	113.67 (16)	C16—C17—H17	120.1
C4—C5—C25	120.92 (17)	C23—C18—C19	119.18 (17)
F2A—C25—F3A	110.1 (10)	C23—C18—C3	120.83 (17)
F1—C25—F3	108.0 (2)	C19—C18—C3	119.79 (16)
F2A—C25—F1A	109.1 (11)	O2—C19—C20	124.45 (19)
F3A—C25—F1A	105.6 (11)	O2—C19—C18	115.52 (16)
F1—C25—F2	106.8 (2)	C20—C19—C18	119.99 (19)
F3—C25—F2	105.34 (18)	C21—C20—C19	119.8 (2)
F2A—C25—C5	108.0 (12)	C21—C20—H20	120.1
F3A—C25—C5	111.9 (12)	C19—C20—H20	120.1
F1—C25—C5	112.64 (17)	C22—C21—C20	121.0 (2)
F3—C25—C5	112.41 (17)	C22—C21—H21	119.5
F1A—C25—C5	112.1 (15)	C20—C21—H21	119.5
F2—C25—C5	111.24 (19)	C21—C22—C23	119.3 (2)
N3—C6—N1	126.45 (15)	C21—C22—H22	120.3
N3—C6—C2	126.26 (16)	C23—C22—H22	120.3
N1—C6—C2	107.28 (15)	C22—C23—C18	120.7 (2)
O1—C7—C8	123.01 (18)	C22—C23—H23	119.7
O1—C7—C4	119.93 (18)	C18—C23—H23	119.7
C8—C7—C4	117.04 (16)	C1—C24—H24A	109.5
C11—C8—C7	128.73 (17)	C1—C24—H24B	109.5
C11—C8—S1	111.23 (15)	H24A—C24—H24B	109.5
C7—C8—S1	120.04 (15)	C1—C24—H24C	109.5
C9—S1—C8	91.27 (11)	H24A—C24—H24C	109.5
C10—C9—S1	113.26 (19)	H24B—C24—H24C	109.5
C10—C9—H9	123.4	O2—C26—H26A	109.5
S1—C9—H9	123.4	O2—C26—H26B	109.5
C9—C10—C11	111.8 (2)	H26A—C26—H26B	109.5
C9—C10—H10	124.1	O2—C26—H26C	109.5
C11—C10—H10	124.1	H26A—C26—H26C	109.5
C8—C11—C10	112.40 (19)	H26B—C26—H26C	109.5
			
C6—N1—N2—C1	0.8 (2)	C3—C4—C7—O1	108.7 (2)
C12—N1—N2—C1	−172.79 (16)	C5—C4—C7—O1	−70.8 (3)
N1—N2—C1—C2	0.5 (2)	C3—C4—C7—C8	−69.6 (2)
N1—N2—C1—C24	−177.61 (19)	C5—C4—C7—C8	110.8 (2)
N2—C1—C2—C3	175.6 (2)	O1—C7—C8—C11	165.9 (2)
C24—C1—C2—C3	−6.5 (4)	C4—C7—C8—C11	−15.8 (3)
N2—C1—C2—C6	−1.5 (2)	O1—C7—C8—S1	−14.5 (3)
C24—C1—C2—C6	176.4 (2)	C4—C7—C8—S1	163.82 (14)
C6—C2—C3—C4	−3.8 (2)	C11—C8—S1—C9	0.30 (18)
C1—C2—C3—C4	179.4 (2)	C7—C8—S1—C9	−179.34 (18)
C6—C2—C3—C18	175.34 (16)	C8—S1—C9—C10	−0.9 (2)
C1—C2—C3—C18	−1.5 (3)	S1—C9—C10—C11	1.3 (3)
C2—C3—C4—C5	1.9 (2)	C7—C8—C11—C10	180.0 (2)
C18—C3—C4—C5	−177.30 (16)	S1—C8—C11—C10	0.4 (2)
C2—C3—C4—C7	−177.68 (16)	C9—C10—C11—C8	−1.0 (3)
C18—C3—C4—C7	3.2 (2)	C6—N1—C12—C13	20.4 (3)
C6—N3—C5—C4	−1.6 (3)	N2—N1—C12—C13	−167.57 (18)
C6—N3—C5—C25	179.31 (16)	C6—N1—C12—C17	−157.6 (2)
C3—C4—C5—N3	1.0 (3)	N2—N1—C12—C17	14.4 (3)
C7—C4—C5—N3	−179.50 (17)	C17—C12—C13—C14	0.3 (3)
C3—C4—C5—C25	180.00 (18)	N1—C12—C13—C14	−177.73 (19)
C7—C4—C5—C25	−0.5 (3)	C12—C13—C14—C15	−0.9 (4)
N3—C5—C25—F2A	78.0 (6)	C13—C14—C15—C16	1.2 (4)
C4—C5—C25—F2A	−101.2 (6)	C14—C15—C16—C17	−0.9 (5)
N3—C5—C25—F3A	−43.4 (6)	C13—C12—C17—C16	0.1 (4)
C4—C5—C25—F3A	137.5 (5)	N1—C12—C17—C16	178.1 (2)
N3—C5—C25—F1	17.1 (3)	C15—C16—C17—C12	0.2 (5)
C4—C5—C25—F1	−162.1 (2)	C4—C3—C18—C23	−63.7 (2)
N3—C5—C25—F3	139.40 (19)	C2—C3—C18—C23	117.2 (2)
C4—C5—C25—F3	−39.7 (3)	C4—C3—C18—C19	111.1 (2)
N3—C5—C25—F1A	−161.8 (5)	C2—C3—C18—C19	−68.0 (2)
C4—C5—C25—F1A	19.0 (6)	C26—O2—C19—C20	10.7 (3)
N3—C5—C25—F2	−102.7 (2)	C26—O2—C19—C18	−167.4 (2)
C4—C5—C25—F2	78.1 (2)	C23—C18—C19—O2	178.74 (17)
C5—N3—C6—N1	178.56 (17)	C3—C18—C19—O2	3.9 (3)
C5—N3—C6—C2	−0.6 (3)	C23—C18—C19—C20	0.6 (3)
N2—N1—C6—N3	178.99 (17)	C3—C18—C19—C20	−174.29 (19)
C12—N1—C6—N3	−8.4 (3)	O2—C19—C20—C21	−178.6 (2)
N2—N1—C6—C2	−1.7 (2)	C18—C19—C20—C21	−0.6 (3)
C12—N1—C6—C2	170.89 (17)	C19—C20—C21—C22	−0.1 (4)
C3—C2—C6—N3	3.4 (3)	C20—C21—C22—C23	0.7 (4)
C1—C2—C6—N3	−178.80 (17)	C21—C22—C23—C18	−0.8 (3)
C3—C2—C6—N1	−175.90 (15)	C19—C18—C23—C22	0.1 (3)
C1—C2—C6—N1	1.87 (19)	C3—C18—C23—C22	174.91 (19)

### Hydrogen-bond geometry (Å, º)

**Table:**

*D*—H···*A*	*D*—H	H···*A*	*D*···*A*	*D*—H···*A*
C13—H13···N3	0.93	2.42	3.012 (2)	122
C11—H11···O1^i^	0.93	2.51	3.114 (3)	122

Symmetry code: (i) −*x*+1/2, *y*+1/2, −*z*+1/2.
