# Supplementary figures and images for: Crystal structure of [4-(2-meth­oxy­phen­yl)-3-methyl-1-phenyl-6-tri­fluoro­methyl-1H-pyrazolo­[3,4-b]pyridin-5-yl](thio­phen-2-yl)methanone
Source: Acta Crystallogr Sect E Struct Rep Online. 2014 Aug 6;70(Pt 9):o974–5. doi: 10.1107/S1600536814017437 (PMC4186079; doi:10.1107/S1600536814017437)

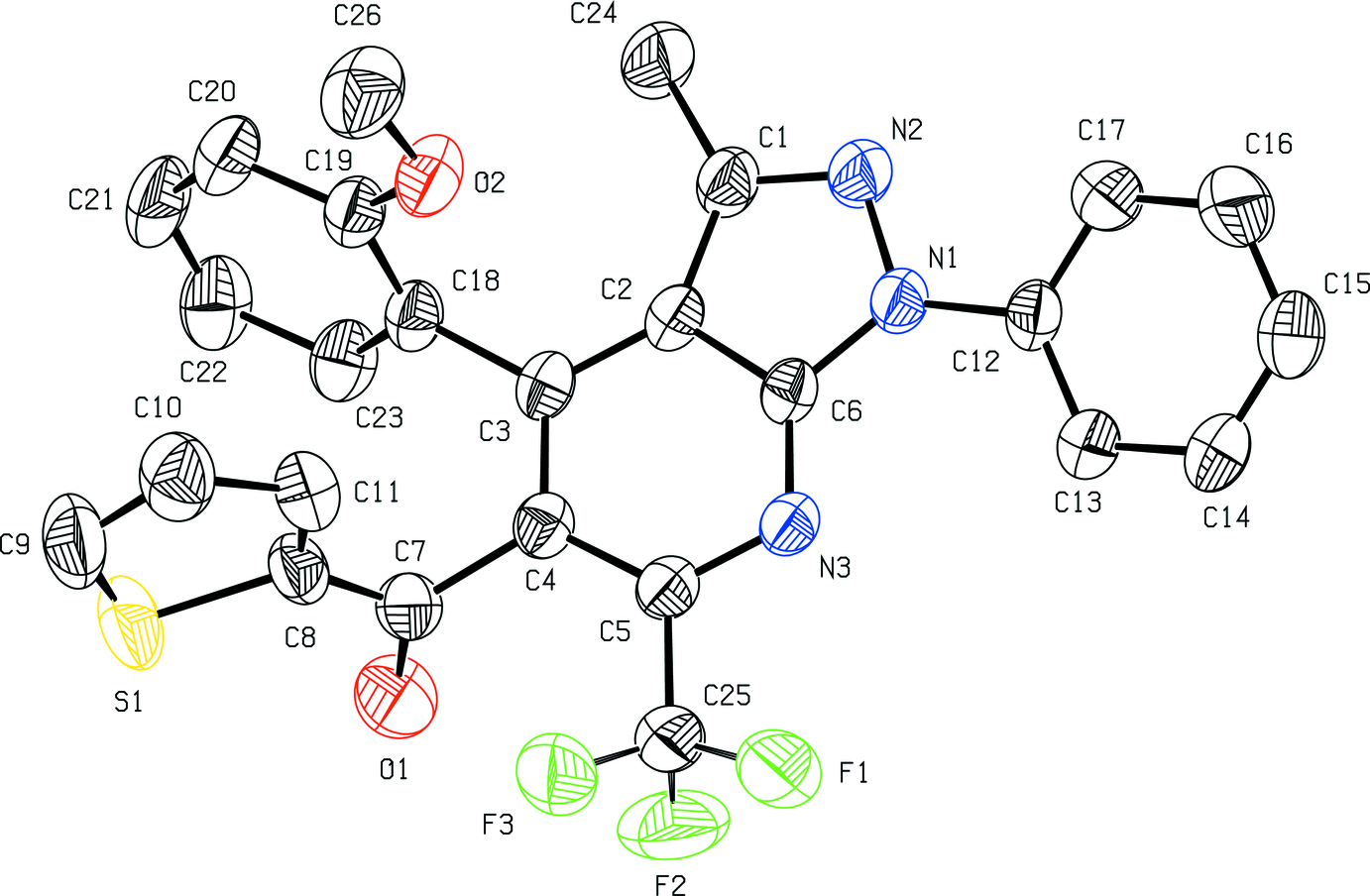

Supplement: Supplementary file 4 [file e-70-0o974-fig1.tif]

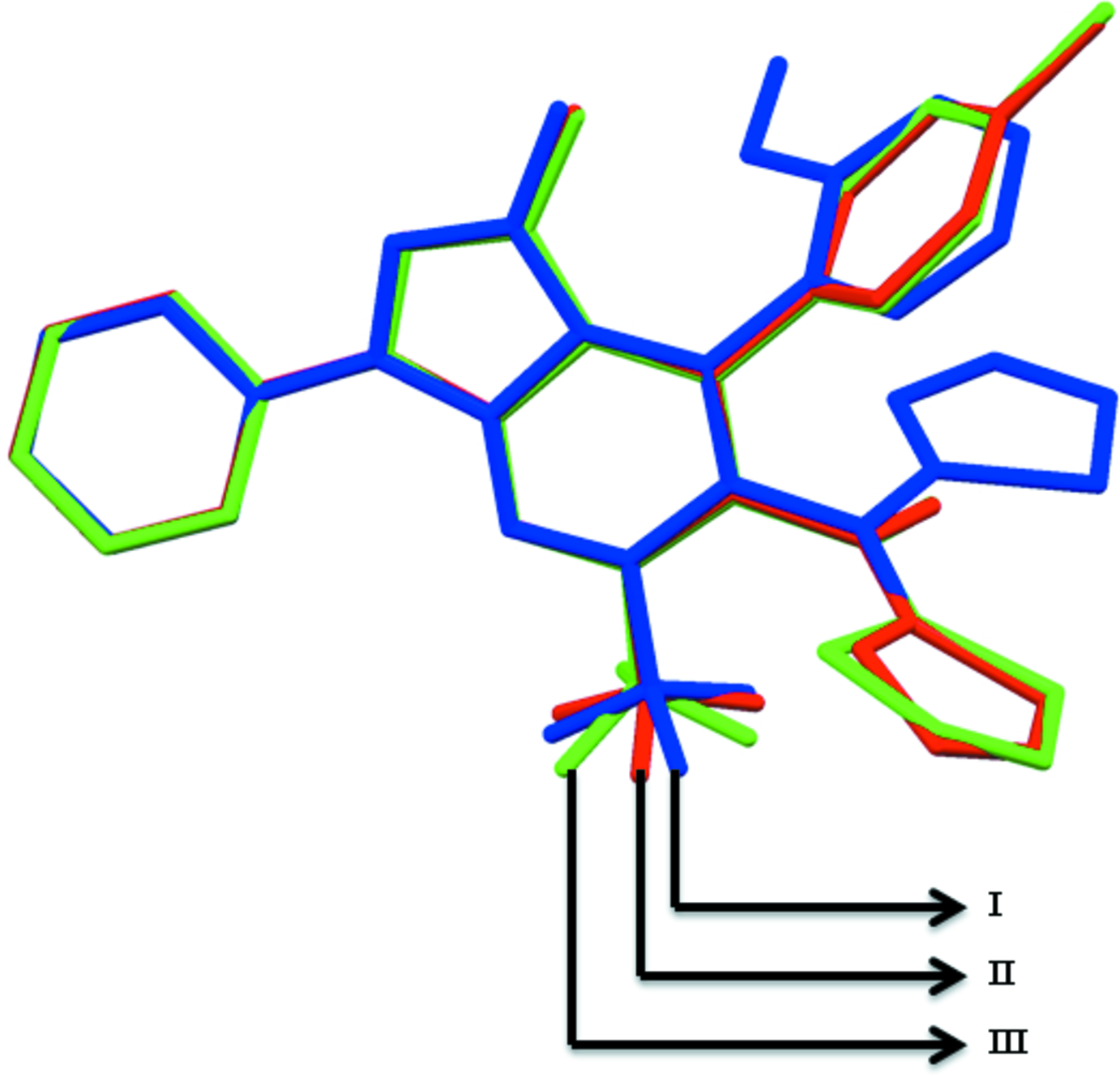

Supplement: Supplementary file 5 [file e-70-0o974-fig2.tif]

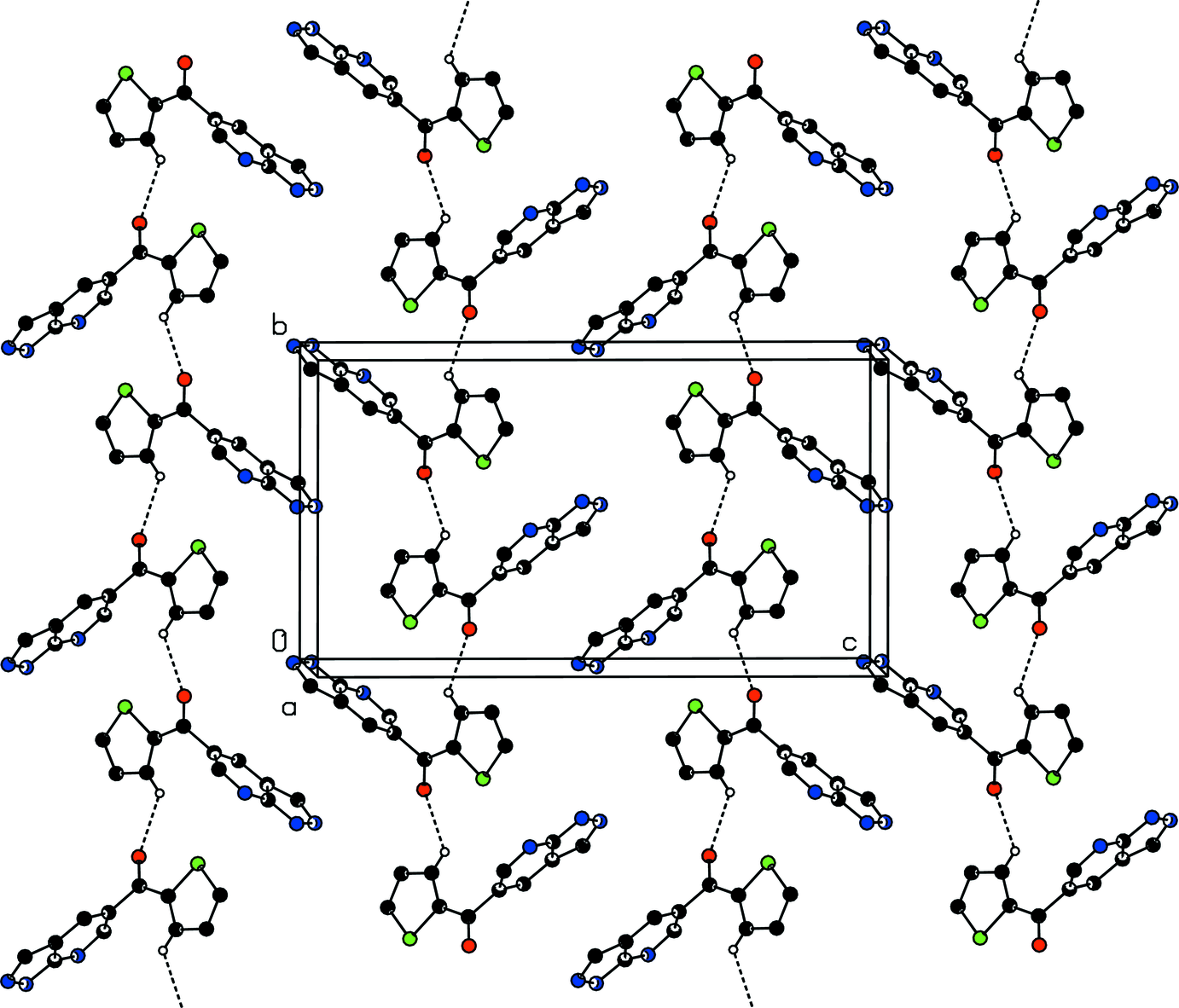

Supplement: Supplementary file 6 [file e-70-0o974-fig3.tif]
